# SOCIAL DETERMINANTS OF CHINESE OLDER ADULTS’ SOCIAL PARTICIPATION: A MULTIPLE-GROUP ANALYSIS

**DOI:** 10.1093/geroni/igae098.3288

**Published:** 2024-12-31

**Authors:** Jianling Liang, Jia Zhuang, Jie Zhuang

**Affiliations:** The Hong Kong Polytechnic University, Hong Kong, Hong Kong; The Hong Kong Polytechnic University, Hong Kong, Hong Kong; The Hong Kong Polytechnic University, Hong Kong, Hong Kong

## Abstract

In China, as the nation’s aging population grows, its family structure diversifies, with more older adults living apart from their children. While social participation is widely acknowledged as a crucial channel for older adults to maintain their social and psychological well-being, its social determinants among older adults from different family structures remain obscure. This study addresses this knowledge gap with data from the Telephone Support Service in Jiangmen City, China (N = 9,871). First, one-way ANOVA results reveal that older adults living alone demonstrate a higher level of social participation than those living with spouses or those with their children (F = 23.441, p <.001). Further, multiple-group analysis (MGA) was performed to determine the group difference across these three elderly groups regarding the relationship between their social participation and health status (i.e., vision, hearing, and mobility) and demographic features (i.e., age and gender). Three group differences were identified. First, compared with those living alone, the relationship between vision and social participation is significantly stronger among older adults living with spouses (CMIN = 4.317, p =.038). Second, the relationship between mobility and social participation among older adults living alone is significantly stronger than that demonstrated among those living with children (CMIN = 5.772, p =.016). Third, the relationship between hearing and social participation is significantly stronger among older adults living alone than those living with children (CMIN = 5.741, p =.017). These findings offer fresh insight to understand the social participation of older adults with different features.

